# Carnosine Content and Its Association with Carnosine-Related Gene Expression in Breast Meat of Thai Native and Black-Bone Chicken

**DOI:** 10.3390/ani11071987

**Published:** 2021-07-02

**Authors:** Panuwat Khumpeerawat, Monchai Duangjinda, Yupin Phasuk

**Affiliations:** 1 Department of Animal Science, Faculty of Agricultural, Khon Kaen University, Khon Kaen 40002, Thailand; khumpeerawat@gmail.com (P.K.); yuplua@kku.ac.th (Y.P.); 2 Network Center for Animal Breeding and Omics Research, Faculty of Agricultural, Khon Kaen University, Khon Kaen 40000, Thailand

**Keywords:** carnosine, *CARNS1*, gene expression, black-bone chicken, Thai native chicken

## Abstract

**Simple Summary:**

Functional meat has become an important issue in the poultry industry because chicken meat is a rich source of carnosine. Additionally, carnosine synthesis is regulated by an ATP-grasp domain-containing protein 1 (*CARNS1*). However, no studies have elucidated the role of carnosine content and *CARNS1* in Thai native and black-bone chicken. Thus, the aim of this study was to select and develop mating programs for functional meat in Thai chicken populations. Our results indicated that different breeds and ages of chickens have different amounts of carnosine and levels of *CARNS1* expression. The carnosine content and *CARNS1* expression decreased linearly as chicken age increased. Moreover, a moderate correlation was observed between carnosine content and *CARNS1* expression levels. Therefore, the *CARNS1* gene could be used to study marker assistant selection in the future.

**Abstract:**

This study aimed to investigate the carnosine content and ATP-grasp domain-containing protein 1 (*CARNS1*) gene expression and their relationship with breast meat of Black Chinese (BC), KU-Phuparn (KP), Pradu Hang Dam (PD), and Black Chinese × Pradu Hang Dam (Sri Mok: SM) to aid in the selection and mating programs for developing functional meat in Thai chicken populations. The results show that the carnosine content in each breed and breed group varied from 428.08 to 553.93 mg/100 g, whereas the relative expression of *CARNS1* ranged from 0.84 to 1.56. The BC and KP chicken breeds had a higher carnosine content (*p* < 0.01) and higher *CARNS1* expression level (*p* < 0.05) than the SM and PD chicken breeds. The carnosine content and relative gene expression for each age ranged from 423.02 to 577.83 mg/100 g and 0.68 to 1.83, respectively. At 4 weeks of age, the carnosine content (*p* < 0.01) and gene expression (*p* < 0.05) were the highest. However, they decreased as chicken age increased further. The carnosine content and gene expression linearly decreased as chicken age increased (*p* < 0.01). The correlation coefficient between the level of gene expression and carnosine content was moderately positive. The results from this study showed that different breeds and ages of chickens have different amounts of carnosine, and *CARNS1* could act as a biomarker to study marker-assisted selection to improve functional meat in the chicken population in Thailand.

## 1. Introduction

Recently, functional meat has become an important issue in the poultry industry worldwide with the increase in consumer demand for high-quality, healthy poultry products. Generally, the skeletal muscles of vertebrate animals have numerous bioactive compounds, especially in chicken meat, which is considered a source of some important bioactive substances such as carnosine [1]. 

Carnosine is an abundant dipeptide in the skeletal muscle and brain of vertebrates which is synthesized from β-alanine and L-histidine by carnosine synthase [2]. It plays an important role in physiological functions such as pH buffering, anti-aging, anti-glycation, anti-inflammation, and antioxidation and has anti-fatigue and neurotransmitter functions [3,4]. Carnosine is used in medicinal applications for treating diseases such as diabetes, Alzheimer’s disease, aging, cancer, and other chronic diseases [5,6]. In addition, it is used in muscle rehabilitation for athletes and the elderly [7,8]. At present, poultry producers aim to increase the carnosine content in chicken meat to meet the consumer demands for healthy food. Several studies have reported that approaches for the development of high nutrient meat such as the supplementation of precursors in the synthesis of carnosine in the feed [9,10] and using breeding programs can be implemented to improve genes related to bioactive compounds [1]. Selection programs have been employed to develop native chicken breeds in numerous Asian countries because the meat quality of the native chicken was popular with consumers and some bioactive substances were higher than those in broiler chickens [11,12]. 

Recently, several studies reported that the carnosine content in chicken breast meat ranged from 130.00 to 798.30 mg/100 g [1,11]. The variations in the amount of carnosine in chicken breast meat differ based on the breed, sex, and age [13,14,15]. Previous studies on broiler breast muscle found that the carnosine synthase 1 (*CARNS1*) gene, also known as ATP-grasp domain-containing protein 1 (*ATPGD1*) gene, is involved in the synthesis of carnosine using ATP [2]. No studies have investigated the expression of *CARNS1* in chicken breast muscles. Thailand is considered a genetically diverse source of chickens among Asian countries. Currently, researchers have attempted to extract benefits from native chickens in the country by producing functional meat to meet the increase in consumers’ demand around the world by focusing on the development of Thai native and black-bone chicken breeds that contain high bioactive substances. 

However, no study has focused on the carnosine content and the expression of *CARNS1* with respect to carnosine syntheses in Thai native and black-bone chicken breeds in Thailand. Thus, the objective of this study was to assess the carnosine content, *CARNS1* expression, and their relationship with breast meat of two black-bone chicken breeds (Black Chinese and KU-Phuparn), one Thai native chicken breed (Pradu Hang Dam), and one crossbred group (Black Chinese × Pradu Hang Dam, or Sri Mok) to aid in the selection and in mating programs for developing functional meat from Thai chicken populations.

## 2. Materials and Methods

### 2.1. Animal Management

This study was approved by the ethics committee of Khon Kaen University (Approval no. IACUC-KKU-35/62). Four genotypes of chicken were evaluated in this study, including the two black-bone chicken breeds (Black Chinese: BC and KU-Phuparn: KP), one native chicken breed (Pradu Hang Dam: PD), and one crossbred group (Black Chinese × Pradu Hang Dam or Sri Mok: SM). One hundred chickens per breed or breed group were used in this study. The housing was an open-air system under typical conditions of Thailand. There were four pens per breed or breed group and 25 chickens per pen (day-old mixed sex). The chickens were fed ad libitum with commercial diets formulated for the native chicken: first period 21% CP, 3200 kcal metabolizable energy (ME)/kg (0–4 weeks), and second period 18% CP, 3200 kcal ME/kg (4–16 weeks) (Table 1).

### 2.2. Tissue Collection

Sixteen chickens (8 male and 8 female chickens) of each breed/or breed group were randomly selected and slaughtered at 4, 8, 12, and 16 weeks of age; thus, a total of 64 samples per breed were used. The breast muscle samples were immediately collected and stored at −80 °C until further laboratory processing and analysis.

### 2.3. Carnosine Extraction and Purification

Extraction and purification were performed using the method described in [14] and [16] with slight modifications. Minced breast meat samples (2.5 g) were homogenized in 10 mL deionized distilled water using a WiseTis HG-15 D homogenizer (Wisd, Witeg, Wertheim, Germany) at 25,000 rpm for 1 min in a 15 mL polyethylene tube and centrifuged at 9989× *g* for 5 min at 4 °C. The supernatant was collected and incubated at 80 °C for 10 min in a temperature-controlled water bath, followed by centrifugation at 9989× *g* for 5 min at 4 °C. Then, 1 mL supernatant was filtered through a nylon syringe filter (0.45 µm, Whatman, Maidstone, UK) into a vial tube of 1.5 mL and stored at −20 °C for HPLC analysis.

### 2.4. HPLC Analysis

An Agilent 1260 Infinity High Performance Autosampler (Agilent Technologies Deutschland GmbH, Waldbronn, Germany), a ZORBAX Eclipse Plus C18 column (4.6 × 150 mm, 5 µm; Agilent, Santa Clara, CA, USA), and a diode array detector (Agilent, Santa Clara, CA, USA) were used for HPLC analysis. Twenty microliters of the extracted solution were injected into the HPLC column with 0.05 M K_2_HPO_4_ as a mobile phase at a flow rate of 0.8 mL/min. The detection was conducted at 211 nm. The content of compounds was calculated using the standard curve for L-carnosine (r^2^ = 0.99) obtained from Sigma-Aldrich Co. (St. Louis, MO, USA).

### 2.5. RNA Extraction

Total RNA was extracted from frozen breast muscle tissue (200 mg) samples. Eight breast muscle tissue samples (from 4 male and 4 female chickens) were randomly selected from each breed or breed group at 4, 8, 12, and 16 weeks of age using the GeneJET RNA Purification Kit (Thermo Fisher Scientific, Waltham, MA, USA) according to the manufacturer’s instructions. The RNA purification and concentration were verified using a spectrophotometer (NanoDrop™ 2000/2000c, Thermo Fisher Scientific, Waltham, MA, USA) where absorbance was measured at 260 and 280 nm. The extracted RNA concentration was at least 100 ng/μL. High-quality RNA should have an A260/A280 ratio greater than 1.9, indicating that the RNA is high purity.

### 2.6. Quantification of mRNA Expression

The one-step quantitative reverse transcription PCR (qRT-PCR) technique was used to measure gene expression level using Bio-Rad CFX96 Touch Real-Time PCR Systems (Bio-Rad, Hercules, CA, USA). The reaction was performed in a volume of 20 μL containing 10 µL of 2× SensiFAST™ SYBR^®^ No-ROX One-Step Mix (Bioline, Taunton, MA, USA), 1 µL (400 nM) of each primer, 0.2 µL reverse transcriptase, 0.4 µL RiboSafe RNase inhibitor, 3.4 µL DEPC-treated water, and 4 µL (20 ng/µL) RNA template. The cycling condition consisted of a cDNA synthesis step at 50 °C for 10 min, a polymerase activation step at 95 °C for 5 min, and then PCR cycling for 40 cycles of 95 °C for 10 s, 58 °C for 25 or 30 s. Melting curves were generated at 65 °C to 95 °C. Each sample was analyzed in duplicate. The relative amount of mRNA expression was expressed as quantification cycle (Cq) values obtained by quantitative real-time PCR (qPCR) analysis and were normalized to the corresponding values of the housekeeping genes (18 S rRNA). The relative gene expression value was calculated using the 2^−ΔΔCT^ method [17] and used the PD breed as the control. Gene-specific primers were designed using Primer-BLAST NCBI, and the primer sequences are described in Table 2.

### 2.7. Statistical Analysis

Carnosine content and the relative gene expression values (2^−ΔΔCT^) were analyzed using the generalized linear model to investigate the effects of breed, age, sex, and all interactions. The impact of age on the gene expression was analyzed by evaluation using orthogonal polynomials, and the correlation between carnosine content and relative gene expression value was analyzed using Pearson correlation (SAS V.9.4, SAS Institute, Cary, NC, USA).

## 3. Results

### 3.1. Carnosine Content in Breast Meat

**Figure 1:**
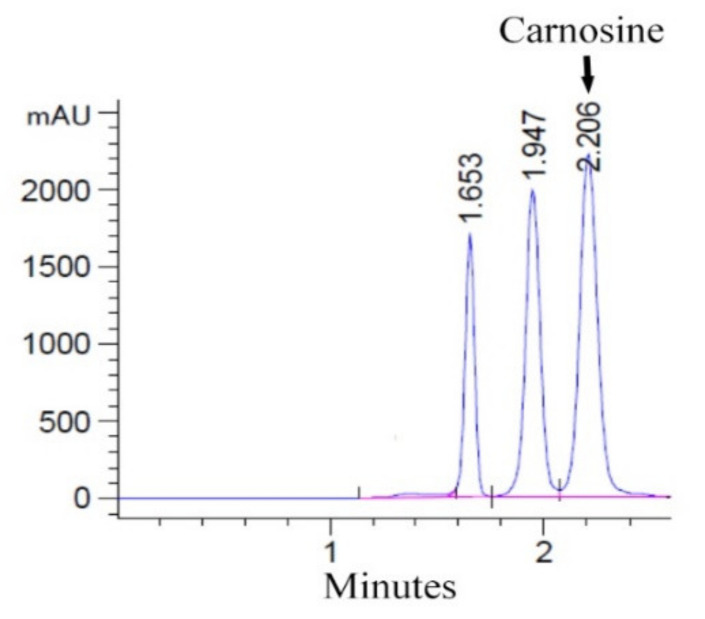
HPLC chromatogram of carnosine extracted from breast muscle samples.

**Figure 2:**
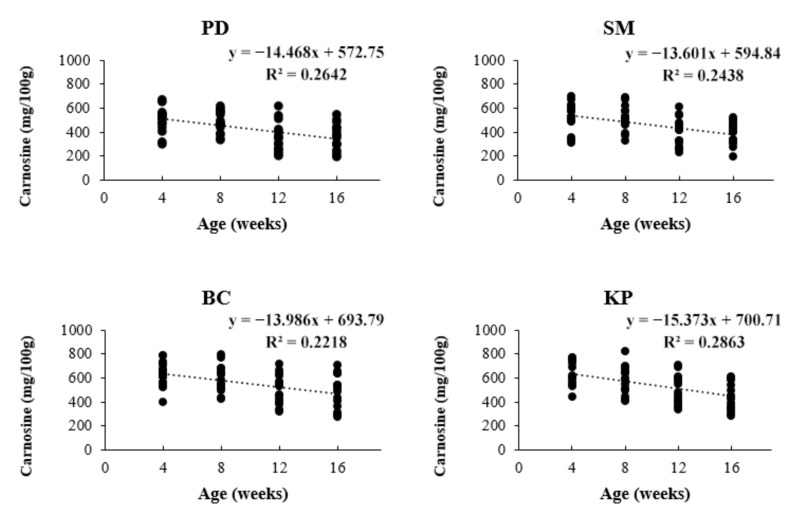
Trends of carnosine content with increasing age. PD = Pradu Hang Dam breed (Thai native chicken); SM = Sri Mok crossbred (crossbred between BC and PD); BC = Black-Chinese breed (black-bone chicken); KP = KU-Phuparn breed (black-bone chicken). A significant negative linearity was observed in this study (*p* < 0.0001).

In this study, the carnosine contents of breast meat analyzed by HPLC (Figure 1) varied from 352.9 to 637.2 mg/100 g (Table 3). All possible interactions among breed, sex, and age did not significantly affect the carnosine content in chicken breast meat (*p* > 0.05). The main factors evaluated in this study showed that the influence of breed and age significantly affected the carnosine content (*p* < 0.001) (Table 3). BC and KP did not differ in terms of the carnosine content; however, their carnosine contents were higher than those of SM and PD (*p* < 0.001). Meanwhile, SM and PD were not different in terms of the carnosine content. Additionally, we studied the effect of age on the carnosine content. At 4 and 8 weeks of age, a higher carnosine content was observed when compared to that at 12 and 16 weeks of age. The carnosine content response decreased as age increased; that is, a negative linearity was observed between carnosine content and age (*p* < 0.0001) (Figure 2).

Mobile phase: 0.05 M K_2_HPO_4_; flow rate: 0.8 mL/min; detection: UV absorbance at 211 nm.

### 3.2. Expression Level of CARNS1

**Figure 3:**
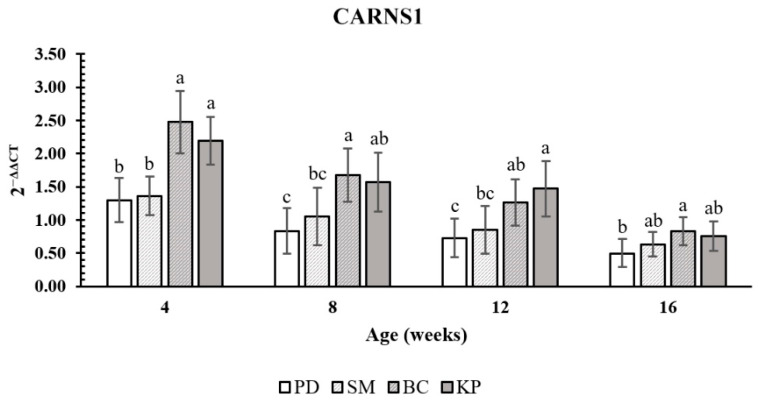
Gene expression level of *CARNS1* in breast meat from Pradu Hang Dam (PD), Sri Mok (SM), Black-Chinese (BC), and KU-Phuparn breeds (KP). ^a,b,c^ Means within a column lacking a common superscript differ (*p* < 0.01).

**Figure 4:**
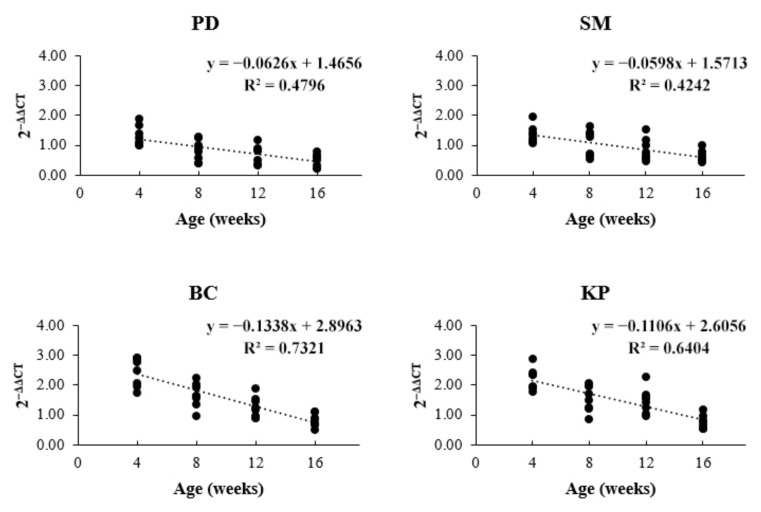
Trend of expression level of *CARNS1* with increasing age. PD = Pradu Hang Dam breed (Thai native chicken); SM = Sri Mok breed (crossbred between BC and PD); BC = Black-Chinese breed (black-bone chicken); KP = KU-Phuparn breed (black-bone chicken). A significant negative linearity was observed in this study (*p* < 0.0001).

In the present study, we evaluated the expression of *CARNS1* in chicken breast meat. The relative expression level of *CARNS1* for carnosine content for the different ages is shown in Figure 3. At 4 and 8 weeks of age, BC had the greatest gene expression level, but it did not differ from that of KP. The gene expression levels of both BC and KP were higher than those of PD and SM. In contrast, at 8 weeks of age, SM was not different from KP. At 12 weeks of age, KP had a higher gene expression level than PD and SM; however, it did not differ from that of BC. At 16 weeks of age, the gene expression level of BC was higher than that of PD, but not different from those of KP and SM. At all weeks of age, PD had the lowest gene expression level, but it did not differ from that of SM. Additionally, the gene expression had a negative linear relationship with increasing age (*p* < 0.0001) (Figure 4).

### 3.3. Correlation between Gene Expression Level and Carnosine Content

**Figure 5:**
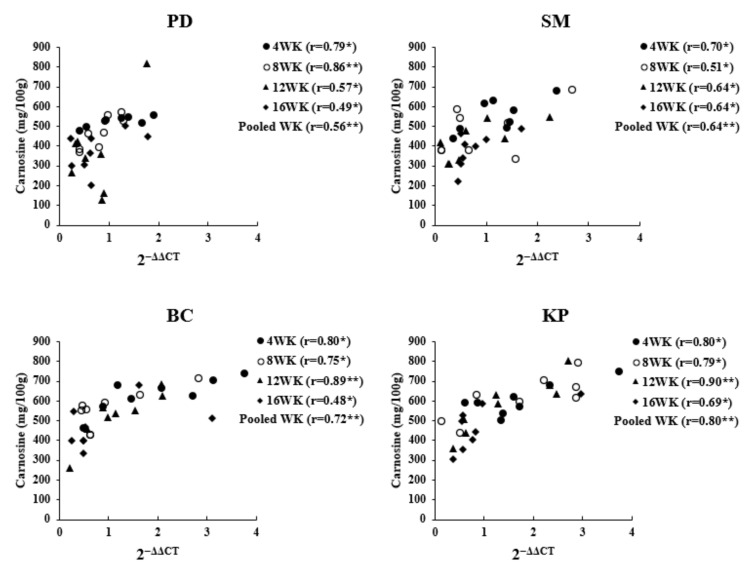
Correlation between carnosine content and expression level of *CARNS1*. PD = Pradu Hang Dam breed (Thai native chicken); SM = Sri Mok breed (crossbred between BC and PD); BC = Black-Chinese breed (black-bone chicken); KP = KU-Phuparn breed (black-bone chicken); 4 WK = 4 weeks of age; 8 WK = 8 weeks of age; 12 WK = 12 weeks of age; 16 WK = 16 weeks of age; r = correlation coefficient: * *p* < 0.05, ** *p* < 0.01.

The results of the correlation analysis show that the expression level of *CARNS1* significantly affected the variability of carnosine content in breast muscle of all chicken breeds used in this study (*p* < 0.01), and the correlation coefficient showed a moderate to high correlation ranging from 0.48 to 0.90 (Figure 5). The expression level of *CARNS1* can explain 48% to 90% of the variance in the carnosine content. Additionally, the correlation coefficient value had a decreasing trend with increasing age.

## 4. Discussion

In this study, we observed higher levels of carnosine content than those observed in previous studies in commercial Korean native chicken [18], 5 lines of Korean native chicken [1], black-bone Silky fowl [14], and broiler chicken [11]. However, the content was lower than that reported in Thai indigenous and hybrid native chickens [13] and Silky fowl [11]. Therefore, this implied that variations in the amount of carnosine may be caused by different genetic backgrounds, which may be a key determinant.

Previously, several studies reported that the carnosine content was affected by breed, sex, and age (13–15). The authors of [11] studied the effects of chicken breed on carnosine content and reported that the Silky fowl—a member of the black-bone chicken—had a higher amount of carnosine when compared to the Japanese native chicken and broiler. In [14], the amount of carnosine in muscle was compared between Silky fowl and White Plymouth Rock; the authors reported that Silky fowl had a higher carnosine content by approximately 2.2-fold than White Plymouth Rock. The results of this study corresponded with those of previous studies, confirming that the black-bone chicken (BC and KP chicken breeds) had higher carnosine content than other breeds used in this study which were raised under the same conditions. Therefore, the breed affected the carnosine content in each chicken breed because of the internal factors involved such as enzymes and transporters in the carnosine synthesis pathway such as carnosine synthase (CARNS), carnosinase-2 (CNDP2), beta-alanine transaminase (ABAT), histidine decarboxylase (HDC), the beta-alanine transporters (TauT ATB0 + and PAT1), and the carnosine or histidine transporters (PEPT1, PEPT2, PHT1 and PHT2) [19] and the composition of muscle fiber types [20]. In [21], the authors reported that type II muscle fiber contains more carnosine than muscle fiber type I. Therefore, further studies are required to elucidate the different biological basis related to carnosine content among the chicken breeds.

Additionally, we showed that increased age affected the carnosine content. Our results were in agreement with [5], who reported that the amounts of carnosine in chicken meat decreased as age increased. Previous studies demonstrated the effects of age on the carnosine content in skeletal muscles. In [20], the authors reported that carnosine content decreased when age increased because of a reduction in the proportion of type II muscle fiber in chicken muscle. Moreover, in [22], the authors observed a strong positive correlation between carnosine content and the type II muscle fiber fraction in human muscle. However, the proportion of muscle fiber type depends on the activities of each age of chicken such as fighting, flying, and feed searching and survival behaviors. These activities increase physical activity, leading to the proliferation of blood capillaries to supply oxygen to muscle fibers, which changes the glycolytic muscle fibers to oxidative muscle fibers, resulting in a change in the muscle fiber type [20,23]. This may be one reason that explains the decline in carnosine levels with increasing age; however, the exact reason remains unclear. As the organisms mature, numerous metabolic changes become involved. 

The effect of sex on the carnosine content in this study agreed with the results of [24], in which it was reported that there was no significant effect of sex on the carnosine content in Korean native chicken meat. Additionally, a previous study reported the same result in a bovine sample [25]. In contrast, Ref. [13] showed that sex had a significant effect on the carnosine content in Thai indigenous and hybrid native chickens and was consistent with the results of [1], who reported that the carnosine content in females was higher than that in males in five lines of the Korean native chicken. Therefore, these results suggest that the effect of sex on the carnosine content in animal skeletal muscle may differ depending on the strain and species. 

The varying gene expression in chicken breeds from this study may be due to the diverse genetic background. Additionally, the increased age affected the expression levels of the *CARNS1* gene. This result demonstrated that this gene played an important function in the early stage of development in chicken muscle. Currently, several studies have reported that age-related gene expression levels are important for the survival of organisms. In [26], it was reported that as the cells of an organism age, a process of homeostasis controls the concentration of reactive oxygen species (ROS) and other metabolites involving numerous metabolic pathways, and these processes are specific to species and tissue, which leads to changes in the gene expression in the regulatory network, which vary with age. Additionally, [27] reported that genetic changes occurring during aging affect an organism’s survival such as DNA damage and telomere shortening, resulting in the alteration of gene expression. Furthermore, [28] reported that epigenetic modifications are regulated by the metabolism process and play a key role in the aging process, thus leading to a difference in gene expression levels during the cell cycle. Numerous aging variables are associated with gene expression levels. However, there is no evidence of age-related expression level of *CARNS1* in chickens. Therefore, further studies are required to investigate the cause of the phenomenon. In addition, no study has reported the relationship between sex and *CARNS1* expression in chickens and other species. Therefore, the result of *CARNS1* expression levels obtained in this study was consistent with the carnosine content level in breast muscle not being different between males and females in the population of chickens used in this study.

Currently, Ref. [29] reported that in a general organism, the concentrations of proteins correlated with the mRNA level were approximately 40%, and the variation in the protein levels in the cell cannot be explained by mRNA levels only. The carnosine synthesis and content in muscle involves many factors including enzymes and protein transporters that are regulated by many genes [19]; in addition, the beta-alanine concentration in the body leads to variations in the mRNA level of the carnosine-synthesis gene [10]. Additionally, gene expression involves the activity rate of cellular processes including transcription, mRNA degradation, and translation, and the protein level depends on the translation and protein degradation rate [30]. Moreover, Ref. [31] reported that the varying ratio of muscle fiber types might influence the variation in the gene expression associated with carnosine synthesis. At present, no study has focused on the correlation of mRNA levels of *CARNS1* and carnosine content in chickens and other species.

## 5. Conclusions

This study showed that breed and age had significant effects on the levels of carnosine content (*p* < 0.01) and *CARNS1* gene expression (*p* < 0.01). BC and KP breeds, which are a group of black-bone chicken breeds, can be considered a superior source of carnosine when compared to SM and PD breeds. In addition, the correlation between carnosine content and *CARNS1* gene expression levels decreased as age increased with a negative linearity. Furthermore, *CARNS1* gene expression was significantly correlated with carnosine content with a moderate to high positive correlation (*p* < 0.01). Therefore, the carnosine content could be possibly employed as an economic trait in the breeding plan, and *CARNS1* could be used as a gene marker to study marker-assisted selection to improve functional meat quality of the chicken population in Thailand.

## Figures and Tables

**Table 1:** Commercial diets formulated for the native chicken.

Details	First Period (0–4 Weeks)	Second Period (4–16 Weeks)
Crude protein (CP) (%)	21	18
Metabolizable energy (ME) (kcal/kg)	3200	3200
Crude fat (%)	3	2
Crude fiber (%)	6	7
Moisture (%)	13	13

**Table 2:** Gene and primer sequences used for qRT-PCR.

Gene	Primer Sequence (5′--> 3′)	Chr	GeneBankID	Product (bp)
*CARNS1*	F: TGCCCTGGAAGAATTTGTGC	5	NM_001172593.1	170
	R: ACAGCAACCAGCGAGAGAG			
18 S rRNA	F: CGGCGACGACCCATTCGAAC	16	XR_003078044.1	99
	R: GAATCGAACCCTGATTCCCCGTC			

**Table 3:** Carnosine content (mg/100 g) (Mean ± SD) of breast meat from Pradu Hang Dam (PD), Sri Mok (SM), Black-Chinese (BC), and KU-Phuparn (KP) chickens at different ages and sexes of chickens.

Breeds	Sexes	Ages (Weeks)				AVG (B) ^1^	SEM	Effect ^2^			
		4	8	12	16			B	A	S	Intac
PD	M	505.5 ± 140.7	490.5 ± 83.6	336.4 ± 87.2	373.0 ± 85.3	428.08 ± 126.9 ^y^					
	F	510.7 ± 51.6	489.8 ± 102.4	369.5 ± 156.4	349.1 ± 134.1						
SM	M	537.9 ± 91.4	521.0 ± 87.9	400.5 ± 135.3	401.9 ± 69.4	458.82 ± 124.2 ^y^					
	F	531.5 ± 138.6	511.7 ± 117.1	375.3 ± 108.0	390.6 ± 115.0						
BC	M	638.6 ± 119.3	593.0 ± 104.3	506.1 ± 131.8	480.2 ± 136.2	553.93 ± 139.8 ^x^					
	F	635.6 ± 94.1	594.8 ± 119.5	501.5 ± 119.7	481.3 ± 159.1						
KP	M	626.7 ± 75.3	592.7 ± 73.9	491.4 ± 153.9	443.6 ± 126.7	546.98 ± 129.5 ^x^					
	F	635.6 ± 124.6	593.4 ± 152.2	527.7 ± 83.8	464.5 ± 96.4						
AVG (A) ^1^		577.8 ± 117.5 ^a^	548.4 ± 111.6 ^a^	438.6 ± 137.5 ^b^	423.0 ± 121.7 ^b^		15.8				
SEM						15.8					
*p*-value								***	***	ns	ns

^1^ AVG (B) = Mean ± SD of breed and breed group; AVG (B) = Mean ± SD of ages; SEM = standard error of mean. ^2^ P = effect of pen, B = breed or breed group, A = age, S = sex, Intac = All levels of interaction (P × B, …, P × B × A × S), ns = not significant differences; *** *p* < 0.001. ^a,b^ Means within a column lacking a common superscript differ (*p* < 0.01); ^x,y^ Means within a row lacking a common superscript differ (*p* < 0.01).

## Data Availability

Additional data are available on request from the corresponding authors.

## References

[1] Jung S., Bae Y.S., Kim H.J., Jayasena D.D., Lee J.H., Park H.B., Heo K.N., Jo C. (2003). Carnosine, anserine, creatine, and inosine 5′-monophosphate contents in breast and thigh meats from 5 lines of Korean native chicken. Poult. Sci..

[2] Drozak J., Veiga-da-Cunha M., Vertommen D., Stroobant V., Van S.E. (2010). Molecular identification of carnosine synthase as ATP-grasp domain-containing protein 1 (ATPGD1). J. Biol. Chem..

[3] Menon K., Mousa A., de Courten B. (2018). Effects of supplementation with carnosine and other histidine-containing dipeptides on chronic disease risk factors and outcomes: Protocol for a systematic review of randomised controlled trials. BMJ.

[4] Caruso G., Fresta C.G., Musso N., Giambirtone M., Grasso M., Spampinato S.F., Merlo S., Drago F., Lazzarino G., Sortino M.A. (2019). Carnosine Prevents Aβ-Induced Oxidative Stress and Inflammation in Microglial Cells: A Key Role of TGF-β1. Cells.

[5] Kim S.K., Kim Y., Baek I.K., Auh J.H. (2012). Carnosine and anserine in chicken: Distribution, age-dependency and their anti-glycation activity. Korean J. Food Sci. Anim. Resour..

[6] Derave W., Courten B.D., Baba S.P. (2019). An update on carnosine and anserine research. Amino Acids.

[7] Baguet A., Bourgois J., Vanhee L., Achten E., Derave W. (2010). Important role of muscle carnosine in rowing performance. J. Appl. Physiol..

[8] del Favero S., Roschel H., Solis M.Y., Hayashi A.P., Artioli G.G., Otaduy M.C., Benatti F.B., Harris R.C., Wise J.A., Leite C.C. (2012). Beta-alanine (CarnosynTM) supplementation in elderly subjects (60–80 years): Effects on muscle carnosine content and physical capacity. Amino Acids.

[9] Kai S., Watanabe G., Kubota M., Kadowaki M., Fujimura S. (2015). Effect of dietary histidine on contents of carnosine and anserine in muscles of broilers. Anim. Sci. J..

[10] Qi B., Wang J., Ma Y., Wu S., Qi G., Zhang H. (2018). Effect of dietary β-alanine supplementation on growth performance, meat quality, carnosine content, and gene expression of carnosine-related enzymes in broilers. Poult. Sci..

[11] Kojima S., Saegusa H., Sakata M. (2014). Histidine-containing dipeptide concentration and antioxidant effects of meat extracts from Silky Fowl: Comparison with meat-type chicken breast and thigh meats. Food Sci. Technol. Res..

[12] Jaturasitha S., Chaiwang N., Kreuzer M. (2016). Thai native chicken meat: An option to meet the demands for specific meat quality by certain groups of consumers; a review. Anim. Prod. Sci..

[13] Intarapichet K.O., Maikhunthod B. (2005). Genotype and gender differences in carnosine extracts and antioxidant activities of chicken breast and thigh meats. Meat Sci..

[14] Tian Y., Xie M., Wang W., Wu H., Fu Z., Lin L. (2007). Determination of carnosine in Black-Bone Silky Fowl (*Gallus gallus domesticus Brisson*) and common chicken by HPLC. Eur. Food Res. Technol..

[15] Ali M., Lee S.Y., Park J.Y., Jung S., Jo C., Nam K.C. (2019). Comparison of functional compounds and micronutrients of chicken breast meat by breeds. Food Sci. Anim. Resour..

[16] Maikhunthod B., Intarapichet K.O. (2005). Heat and ultrafiltration extraction of broiler meat carnosine and its antioxidant activity. Meat Sci..

[17] Livak K.J., Schmittgen T.D. (2001). Analysis of relative gene expression data using real-time quantitative PCR and the 2(−ΔΔC(T)) Method. Methods.

[18] Jayasena D.D., Jung S., Bae Y.S., Kim S.H., Lee S.K., Lee J.H., Jo C. (2014). Changes in endogenous bioactive compounds of Korean native chicken meat at different ages and during cooking. Poult. Sci..

[19] Everaert I., Naeyer H.D., Taes Y., Derave W. (2013). Gene expression of carnosine-related enzymes and transporters in skeletal muscle. Eur. J. Appl. Physiol..

[20] Lokman I.H., Jawad H.S.A., Goh Y.M., Sazili A.Q., Noordin M.M., Zuki A.B.Z. (2016). Morphology of breast and thigh muscles of Red Jungle Fowl (*Gallus gallus spadiceus*), Malaysian village chicken (*Gallus gallus domesticus*) and commercial broiler chicken. Int. J. Poult. Sci..

[21] Boldyrev A.A., Aldini G., Derave W. (2013). Physiology and pathophysiology of carnosine. Physiol. Rev..

[22] Baguet A., Everaert I., Hespel P., Petrovic M., Achten E., Derave W. (2011). A new method for non-invasive estimation of human muscle fiber type composition. PLoS ONE.

[23] Ismail I., Joo S.T. (2017). Poultry meat quality in relation to muscle growth and muscle fiber characteristics. Korean J. Food Sci. Anim. Resour..

[24] Jayasena D.D., Jung S., Kim S.H., Kim H.J., Alahakoon A.U., Lee J.H., Jo C. (2015). Endogenous functional compounds in Korean native chicken meat are dependent on sex, thermal processing and meat cut. J. Sci. Food Agri..

[25] Mateescu R.G., Garmyn A.J., O’Neil M.A., Tait R.G., Abuzaid A., Mayes M.S., Garrick D.J., Van Eenennaam A.L., VanOverbeke D.L., Hilton G.G. (2012). Genetic parameters for carnitine, creatine, creatinine, carnosine, and anserine concentration in longissimus muscle and their association with palatability traits in Angus cattle. J. Anim. Sci..

[26] Brink T.C., Demetrius L., Lehrach H., Adjaye J. (2009). Age-related transcriptional changes in gene expression in different organs of mice support the metabolic stability theory of aging. Biogerontology.

[27] Cararo J.H., Streck E.L., Schuck P.F., Ferreira G.C. (2015). Carnosine and related peptides: Therapeutic potential in age-related disorders. Aging Dis..

[28] Ren R., Ocampo A., Liu G.H., Izpisua B.J.C. (2017). Regulation of stem cell aging by metabolism and epigenetics. Cell Metab..

[29] Vogel C., Marcotte E.M. (2012). Insights into the regulation of protein abundance from proteomic and transcriptomic analyses. Nat. Rev. Genet..

[30] Schwanhäusser B., Busse D., Li N., Dittmar G., Schuchhardt J., Wolf J., Chen W., Selbach M. (2011). Global quantification of mammalian gene expression control. Nature..

[31] Cong J., Zhang L., Li J., Wang S., Gao F., Zhou G. (2017). Effects of dietary supplementation with carnosine on growth performance, meat quality, antioxidant capacity and muscle fiber characteristics in broiler chickens. J. Sci. Food Agric..

